# Assessing racial differences in time to subsequent treatment following androgen deprivation therapy among Veterans with prostate cancer

**DOI:** 10.1038/s41391-025-00995-4

**Published:** 2025-07-04

**Authors:** Nadine A. Friedrich, Jessica L. Janes, Joshua Parrish, Amanda M. De Hoedt, Janis Pruett, Mark Fallick, Raj Gandhi, Agnes Hong, Nicholas P. Tatonetti, Stephen J. Freedland

**Affiliations:** 1 Department of Urology, Samuel Oschin Comprehensive Cancer Institute, Cedars-Sinai Medical Center, Los Angeles, CA USA; 2 Department of Computational Biomedicine, Cedars-Sinai Medical Center, Los Angeles, CA USA; 3 Urology Section, Department of Surgery, Veterans Affairs Health Care System, Durham, NC USA; 4 Brisbane, CA USA; 5 Pfizer Inc, New York, NY USA; 6 Cedars-Sinai Cancer, Cedars-Sinai Medical Center, Los Angeles, CA USA

**Keywords:** Cancer therapy, Outcomes research

## Abstract

**Background:**

For metastatic and certain advanced prostate cancer (PC), guidelines support intensified androgen deprivation therapy (ADT) as first-line (1 L) systemic treatment. However, some patients receive ADT alone, leading to tumor progression requiring 2^nd^ line therapy. Despite racial disparities in PC outcomes, there are no population-level studies assessing racial differences in time to subsequent treatment after 1 L ADT.

**Methods:**

We performed a retrospective population-level analysis to assess the association between race and time to subsequent treatment after ADT in the Veterans Affairs Health Care System. Primary outcome was time from ADT monotherapy to subsequent treatment, defined as receipt of androgen receptor pathway inhibitor (ARPI), non-steroidal first-generation anti-androgen (NSAA), chemotherapy, or other treatments. We used Cox competing risks models and Kaplan-Meier (KM) analyses to estimate subsequent treatment rates by Non-Hispanic White [NHW], Non-Hispanic Black [NHB], Hispanic and Other patients, adjusted for baseline covariates.

**Results:**

From 2001–2021, 141,495 PC patients received ADT alone. During median (IQR) follow-up of 51.1 (22.8, 97.2) months, 28,144 patients (20%) had subsequent treatment: 11,319 (40%) ARPIs, 12,990 (46%) NSAAs, 3402 (12%) chemotherapy and 433 (2%) other 2^nd^ line therapies. NHB had significantly lower subsequent treatment rates (HR = 0.82, 95% CI = 0.80–0.85) vs. NHW. Both Hispanic (HR = 0.93, 95%CI = 0.88–0.98) and Other men (HR = 0.91, 95%CI = 0.84–0.98), also had lower subsequent treatment rates. When stratified by age, associations between race/ethnicity and time to subsequent treatment were stronger in younger patients.

**Conclusions:**

All races examined had significantly lower rates of subsequent treatment after 1 L ADT relative to NHW, especially in younger patients. Further investigation is needed to determine if these lower rates of subsequent treatment reflect lower rate of progression or undertreatment of progressing patients.

## Introduction

Advanced and metastatic prostate cancer (PC) is often treated with androgen deprivation therapy (ADT) [1], which decreases PSA and slows tumor growth. However, eventually, many patients will progress to castration-resistant PC (CRPC) either as rising PSA or radiographic progression and require a second line of systemic therapy [2]. Guideline concordant options for CRPC, depending on tumor metastasis, include chemotherapy and androgen receptor pathway inhibitors (ARPIs; such as abiraterone, apalutamide, darolutamide, and enzalutamide) [3, 4]. Other options, like Radium-223 and Sipuleucel-T, are used infrequently as first-line CRPC agents [5]. Some men with CRPC are also treated with first-generation non-steroidal anti-androgens (NSAA) (i.e., bicalutamide), despite not being guideline concordant [6]. Regardless of the agent used, subsequent treatment following ADT alone typically indicates tumor progression and poor prognosis [7].

While Black men are more likely to be diagnosed with PC and are nearly two times more likely to die from PC versus white men [8], whether time to subsequent treatment following ADT initiation differs by race (Non-Hispanic White [NHW], Non-Hispanic Black [NHB], Hispanic and Other) is unknown. To examine this, we used the Veterans Affairs (VA) health care system (VAHCS) data to perform a retrospective population-level analysis assessing the association between race and subsequent treatment after ADT. Time to subsequent treatment was analyzed as a key clinical endpoint, serving as a proxy for disease progression that requires the next line of therapy. In clinical trials evaluating first-line systemic treatments for PC, time to subsequent treatment has been used as a key secondary endpoint to measure treatment efficacy [9]. Based on previous studies from our team, which showed no difference in time to metastasis after ADT [10], we hypothesized that time to subsequent treatment would be similar across races.

## Materials and methods

### Design & cohort

After IRB approval, we performed a nationwide retrospective study using the VA Informatics and Computing Infrastructure (VINCI), an analytic platform with access to all electronic health record (EHR) data in the Veterans Affairs Health Care System. The VA is the largest integrated, federally funded healthcare network in the United States, comprising tertiary care centers, regional hospitals, and outpatient clinics. We utilized specific queries within VINCI to identify male veterans who were diagnosed with PC between 2001 and 2021 and were ≥18 years old at diagnosis. We limited queries to individuals who were considered active VA users (defined as ≥2 visits within the 5 years of the study period). Demographic and clinical data were extracted from VINCI, including first receipt of each of the following PC-specific treatments: bilateral orchiectomy, radiation, radical prostatectomy, ADT, ARPIs (abiraterone, apalutamide, darolutamide, and enzalutamide), NSAAs (bicalutamide, flutamide, and nilutamide), chemotherapy (cabazitaxel, docetaxel), and other systemic therapies (olaparib, pembrolizumab, radium-223, sipuleucel-T).

**Fig. 1:**
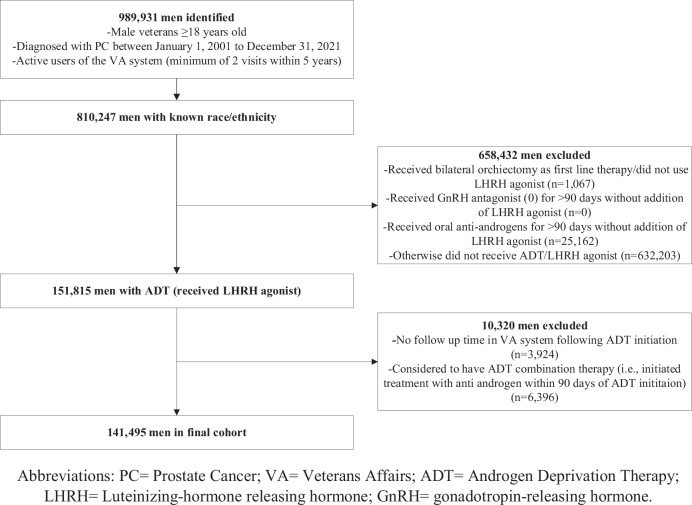
Consort diagram.

Only patients who received ADT, defined as luteinizing-hormone releasing hormone (LHRH) agonist (Leuprolide, Goserelin, Triptorelin), as 1 L systemic therapy for PC were included. The number of men who received LHRH antagonists was small and therefore these patients were excluded. Patients may have received prior local therapy for PC. Patients may also have received an NSAA for up to 90 days for blocking of testosterone flare, but patients who stayed on NSAA for ≥90 days were considered on combined androgen blockade and were excluded. We also excluded patients who received a second systemic treatment initiated within 90 days after ADT start. Patients were excluded if they were missing pertinent data on race or ethnicity or had no follow-up after ADT start (Fig. 1).

### Study variables

Race and ethnicity were both self-reported and combined into a single variable with categories of NHW, NHB, Hispanic (regardless of race), or Other. Other included Asian, Biracial, Native Hawaiian/Other Pacific Islander, and Native American/Alaskan Native races of Non-Hispanic ethnicity. Age at ADT and year of ADT were captured and treated as continuous variables. Body Mass Index (BMI) was derived using the weight closest to, but prior to ADT initiation, and median height across all heights observed. BMI was categorized as <25, 25–29, ≥30, or unknown if missing either height or weight. Comorbidities defined under the Charlson Comorbidity Index (CCI) were identified from claims data (ICD 9/10 codes) and summed to the day before the ADT start (index date). CCI was categorized as 0, 1, 2, or 3 + . Clinical stage and Gleason grade were obtained from the cancer registry, these data were absent for most patients and, therefore, not used in the analysis. All PSA and testosterone measurements were captured, excluding values above or below 3 standard deviations. Baseline PSA and testosterone variables were identified as the value closest to but prior to ADT start. Both PSA and testosterone were captured as continuous variables but analyzed as categorical variables to include those with unknown values. PSA was categorized as <4, 4–10, 10.1–19.9, ≥20, or unknown, and testosterone was categorized into quartiles or unknown. Receipt of radiation or radical prostatectomy prior to ADT was both categorized as yes versus no. Number of months from PC diagnosis to ADT initiation was computed.

Our primary outcome was time from ADT to subsequent treatment, defined as (1) receipt of add-on ARPI therapy (abiraterone, apalutamide, darolutamide, enzalutamide) or other systemic 2^nd^ line therapy (Olaparib, Pembrolizumab, Radium-223, Sipuleucel-T), (2) addition of a NSAA therapy, or (3) receipt of chemotherapy (cabazitaxel, docetaxel). If patients received multiple treatments, earliest date was used. Patients with no evidence of subsequent treatment were censored at time of death or last known visit within the VA system.

### Statistical analysis

Baseline demographic and clinical characteristics were summarized at time of ADT using median, interquartile range, and range for continuous variables and frequencies and percentages for categorical variables. Differences in characteristics between race/ethnicity groups were assessed using Kruskal-Wallis tests for continuous variables and Chi square tests or Fisher’s Exact tests where appropriate for categorical variables.

Kaplan-Meier (KM) curves for time from ADT to subsequent treatment were stratified by race/ethnicity and point estimates for the proportion free from subsequent treatment at 3, 5, 7, 10, and 15 years post-ADT were obtained. A log-rank test was used to test for differences in curves between groups. Univariable and multivariable cause-specific Cox proportional hazards models assessed the association between race/ethnicity and time to subsequent treatment, treating death from all causes as a competing risk. Candidate variables for inclusion in the multivariable models included race/ethnicity, age at ADT, year of ADT, time from PC diagnosis to ADT, CCI, BMI, PSA and testosterone levels prior to ADT, radiation therapy, and radical prostatectomy prior to ADT. Variables that were selected a priori in univariable analysis were included in the multivariable model. We followed the rule of thumb to only include 1 predictor for every 10 events observed to reduce the likelihood of overfitting. If the model needed to be reduced, comparisons between variations of the model were assessed with model fit indices such as Bayesian information criterion (BIC) and Akaike information criterion (AIC) to determine the best fitting model.

Collinearity between variables in the multivariable model was checked and variance inflation factors were assessed. Assumptions of linearity and proportional hazards were assessed with standard methods such as plotting Martingale and Schoenfeld residuals by time. Non-linearity with year of ADT was explored with the use of restricted cubic splines; regardless of the number and placement of knots, effects remained essentially identical to when year was treated as linear, and thus the linear version was kept in the final model. The final model included all candidate variables except prior receipt of radical prostatectomy which was collinear with PSA and added no additional predictive value. Interactions between race/ethnicity and covariates were tested in multivariable analysis. Due to a significant interaction between race/ethnicity and age at ADT, we also stratified associations between race/ethnicity and time to subsequent treatment by age groups ( < 60, 60–69, 70–79, ≥80). For exploratory purposes, the number of treatment events in each group were categorized and reported by treatment type (ARPI therapy, other systemic 2^nd^ line therapy, NSAA, or chemotherapy) among those treated.

We did not include stage (or grade) in our analysis given that most patients were missing this information from the cancer registry. Nonetheless, we conducted a sensitivity analysis limited to 14,538 (10%) patients who had stage and grade data available from the cancer registry to assess associations between race/ethnicity and time to treatment escalation after considering disease severity. For this analysis, we excluded patients with “other” races as cell sizes were very small and would not yield reliable results. We repeated the same univariable and multivariable analyses described above, but in the multivariable model, adjusted for T stage, M stage, and Grade Group. To assess whether the relationship between race and treatment escalation varied by stage, we also stratified the multivariable analysis by M stage and tested the interaction between race and M stage.

All statistical analyses were performed with SAS Enterprise Guide 8.2. Statistical significance was predetermined at *p* < 0.05.

## Results

A total of 989,931 PC patients were identified, of which 810,247 had known race and ethnicity. Among these, 151,815 (19%) received ADT as a 1 L systemic therapy at some point during their PC journey, of whom 145,419 received ADT as monotherapy in 1 L. After excluding those who initiated ADT on the same date as their last known visit to the VA, the study cohort consisted of 141,495 patients. Of this cohort, 94,500 (67%) were NHW, 36,421 (26%) NHB, 7287 (5%) Hispanic, and 3,287 (2%) Other.

### Patient baseline demographics and clinical characteristics by race/ethnicity

NHB patients were the youngest at ADT initiation [Median (Q1–Q3) = 68.1 (62.3–74.8)] (*p* < 0.001), received ADT in later years of the study [Median (Q1–Q3) = 2012 (2007–2017)] (*p* < 0.001), had the highest PSA levels prior to ADT initiation [Median (Q1–Q3) = 10.4 (5.2–25.2)] (*p* < 0.001), the highest number with 3 or more comorbidities prior to ADT initiation (46%) (*p* < 0.001), and the highest rate of radiation use prior to ADT (10%)(*p* < 0.001) compared to all other groups **(**Table 1**)**. Hispanic patients had the highest testosterone levels prior to ADT initiation [Median (Q1–Q3) = 315 (210–424)] (*p* < 0.001), the highest proportion of those with BMI range 25–29 (42%) (*p* < 0.001), and the longest follow-up [Median (Q1–Q3) = 58.4 (25.2, 109.0)] (*p* < 0.001) compared to all other groups.

**Table 1:** Characteristics of patients who received ADT stratified by race/ethnicity (*N* = 141,495).

	Non-Hispanic White (*N* = 94,500)	Non-Hispanic Black (*N* = 36,421)	Hispanic (*N* = 7287)	Other (*N* = 3287)	*p* value
Age at ADT start					<0.001
Median	73.0	68.1	73.4	71.5	
Q1, Q3	66.9, 79.8	62.3, 74.8	67.1, 79.2	65.4, 78.0	
Range	(36.2–104.2)	(38.1–104.9)	(39.7–99.8)	(39.2–101.7)	
Year of ADT start					<0.001
Median	2011	2012	2011	2012	
Q1, Q3	2006, 2016	2007, 2017	2006, 2016	2007, 2017	
Range	(2001–2022)	(2001–2022)	(2001–2022)	(2001–2021)	
PSA (ng/ml) at ADT start					<0.001
Missing	13928	3228	719	484	
Median	7.9	10.4	8.2	8.5	
Q1, Q3	3.3, 18.2	5.2, 25.2	4.0, 18.3	4.0, 19.2	
Range	(0.0–614.0)	(0.0–614.0)	(0.0–606.7)	(0.0–610.0)	
Testosterone level at ADT start					<0.001
Missing	84,401	31,036	6444	2883	
Median	281.3	301.0	315.0	288.1	
Q1, Q3	174.0, 410.0	200.0, 428.0	210.0, 424.0	163.5, 408.9	
Range	(0.0–5800.0)	(0.0–1500.0)	(0.0–1419.0)	(1.6–1500.0)	
CCI at ADT start					<0.001
0	26,148 (28%)	8125 (22%)	1627 (22%)	797 (24%)	
1	18,638 (20%)	6870 (19%)	1464 (20%)	640 (19%)	
2	13,114 (14%)	4773 (13%)	976 (13%)	467 (14%)	
3+	36,600 (39%)	16,653 (46%)	3220 (44%)	1383 (42%)	
BMI at ADT start					<0.001
Missing	16,362	5532	1196	611	
<25	18,999 (24%)	9292 (30%)	1745 (29%)	736 (28%)	
25-29	30,854 (39%)	10770 (35%)	2541 (42%)	1021 (38%)	
≥30	28,285 (36%)	10827 (35%)	1805 (30%)	919 (34%)	
Months from PC diagnosis to ADT start					<0.001
Median	2.8	3.0	3.0	3.0	
Q1, Q3	0.8, 21.4	1.1, 15.3	1.0, 14.9	0.9, 17.9	
Range	(0.0–250.0)	(0.0–243.8)	(0.0–234.4)	(0.0–246.1)	
RP prior to ADT start?					<0.001
Yes	4573 (5%)	2329 (6%)	452 (6%)	169 (5%)	
Radiation prior to ADT start?					<0.001
Yes	6278 (7%)	3584 (10%)	610 (8%)	261 (8%)	
Follow up*					<0.001
Median	49.4	53.8	58.4	50.9	
Q1, Q3	21.9, 94.5	24.8, 101.4	25.2, 109.0	23.9, 99.1	
Range	(0.0–262.0)	(0.0–261.7)	(0.0–260.8)	(0.0–259.4)	

*Number of months from ADT to subsequent treatment or to censor date if no subsequent treatment. Across all patients the median (Q1, Q3) was 51.1 (22.8–97.2) months. Overall follow-up to death or censor date was 59.6 (29.3-107.0) months. *ADT* Androgen Deprivation Therapy, *PSA* Prostate Specific Antigen, *CCI* Charlson Comorbidity Index, *BMI* Body Mass Index, *PC* Prostate Cancer, *RP* Radical Prostatectomy.

### Time to subsequent treatment and KM estimates by race/ethnicity

**Fig. 2:**
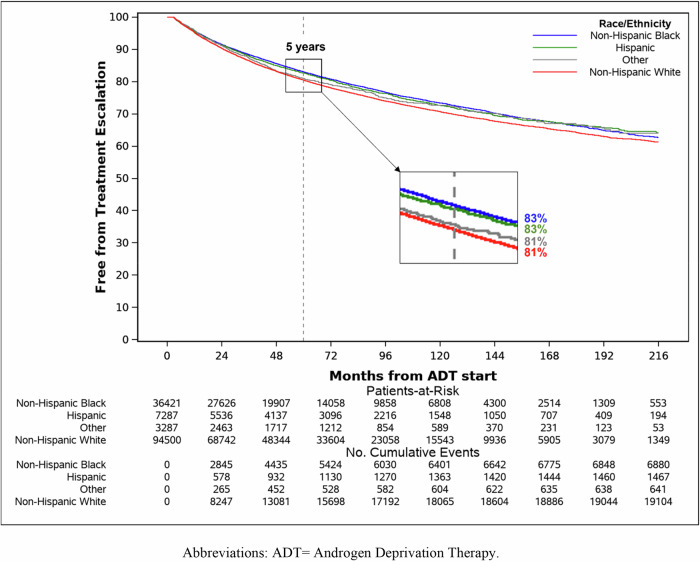
Kaplan-Meier curve for time to subsequent treatment stratified by race.

With a median (Q1–Q3) follow-up of 51.1 (22.8–97.2) months, 28,144 (20%) subsequent treatment events were observed across all races. Among NHWs, 19,133/94,500 (20.2%) events were observed compared to 6900/36,421 (18.9%) for NHBs, 1468/7287 (20.1%) for Hispanics, and 643/3287 (19.6%) for Others **(**Table 2**)**. NHWs were most likely to have subsequent treatment over time (log-rank *p*-value < 0.001) **(**Fig. 2**)**. The 3- and 5-year estimates (95% CI) of being subsequent treatment-free were 86.5% (86.2–86.7%) and 80.5% (80.2–80.8%), respectively for NHWs compared to 88.4% (88.0–88.7%) and 83.0 (82.6–83.5%), respectively for NHBs.

**Table 2:** Subsequent treatment events (28,144/141,495) and Kaplan-Meier point estimates for the percent free from subsequent treatment by race/ethnicity.

Race/Ethnicity	Event/Total	Point Estimates (95% CI)				
		3 years	5 years	7 years	10 years	15 years
Non-Hispanic White	19,133/94,500	86.5 (86.2–86.7%)	80.5 (80.2–80.8%)	76.0 (75.6–76.3%)	70.7 (70.3–71.1%)	64.2 (63.7–64.8%)
Non-Hispanic Black	6900/36,421	88.4 (88.0–88.7%)	83.0 (82.6–83.5%)	78.7 (78.2–79.2%)	73.4 (72.8–74.0%)	66.3 (65.4–67.2%)
Hispanic	1468/7287	87.9 (87.1–88.7%)	82.6 (81.7–83.6%)	78.0 (76.9–79.2%)	72.6 (71.3–74.0%)	66.4 (64.7–68.2%)
Other	643/3287	87.3 (86.1–88.6%)	81.0 (79.5–82.6%)	77.4 (75.6–79.1%)	72.7 (70.7–74.8%)	66.6 (63.8–69.5%)

Note: the KM median was not reached for any group during the study period.

### Univariable and multivariable associations with time to subsequent treatment

In univariable analysis, NHBs experienced significantly lower rates of subsequent treatment [HR (95% CI): 0.89 (0.86, 0.91)] compared to NHWs, as did Hispanics [HR (95% CI): 0.90 (0.86, 0.95)] (Table 3). Others were also at lower rate than NHWs but not significantly so [HR (95% CI): 0.94 (0.87, 1.01)]. In multivariable analysis, all races were at significantly lower rates for escalating treatment compared to NHWs, including NHBs [HR (95% CI): 0.82 (0.80, 0.85)], Hispanics [HR (95% CI): 0.93 (0.88, 0.98)], and Others [HR (95% CI): 0.91 (0.84, 0.98)]. However, subsequent treatment was lowest in NHBs (lowest HR relative to NHWs).

**Table 3:** Univariable and multivariable associations between race/ethnicity and time to subsequent treatment (*N* = 141,495).

Variable	Univariable			Multivariable*		
	HR	95% CI	*p*-value	HR	95% CI	*p*-value
Race/Ethnicity						
Non-Hispanic White		Ref.			Ref.	
Non-Hispanic Black	0.89	(0.86, 0.91)	<0.001	0.82	(0.80, 0.85)	<0.001
Hispanic	0.90	(0.86, 0.95)	<0.001	0.93	(0.88, 0.98)	0.006
Other	0.94	(0.87, 1.01)	0.109	0.91	(0.84, 0.98)	0.015

*Multivariable model adjusted for age, year of ADT start, months from PC diagnosis to ADT start, CCI, PSA, testosterone level, BMI, and prior radiation treatment. *ADT* Androgen Deprivation Therapy, *PSA* Prostate Specific Antigen, *CCI* Charlson Comorbidity Index, *BMI* Body Mass Index, *PC* Prostate Cancer.

When multivariable results were stratified by age groups (< 60, 60-69, 70-79, ≥80), the association between race/ethnicity and time to subsequent treatment was stronger in those with younger ages and weaker in those with older ages (Supplemental Table 1). For example, the HR (95% CI) for NHBs vs. NHWs was 0.75 (0.69, 0.81) in those aged <60 years, 0.79 (0.75, 0.82) in those aged 60–69 years, 0.83 (0.79, 0.87) in those aged 70–79 years, and 0.99 (0.93, 1.06) in those who were 80 years or older. While the effects for Hispanics and Others vs. Non-Hispanic Whites also appeared to vary with age, they did not do so in a consistent fashion (as observed with Non-Hispanic Blacks). When multivariable results included year of ADT as a spline variable with 4 knots placed at the quartiles, effects for race/ethnicity were nearly identical to when year of ADT was treated as linear (Supplemental Table 2).

### Subsequent treatment events broken down by type of treatment category

Among all 28,144 subsequent treatment events, 12,990 (46%) were NSAA, 11,319 (40%) were ARPIs, 3402 (12%) were chemotherapy, and 433 (2%) were other systemic 2^nd^ line therapies **(**Table 4**)**. While the rate of ARPIs between NHBs and NHWs was similar (41% vs. 40%, respectively), Hispanics had the lowest proportion (35%). Hispanics had the highest (51%) proportion of events that were NSAA followed by NHWs (47%), while NHBs were most likely to get chemotherapy (15%) as subsequent treatment, followed by Hispanics (13%) and NHWs (11%).

**Table 4:** Subsequent treatment events (*N* = 28,144) broken down by type of treatment category.

Subsequent treatment Type	Overall (*N* = 28,144)		Non-Hispanic White (19,133)		Non-Hispanic Black (6900)		Hispanic (1468)		Other (*N* = 643)	
	*n*	%	*n*	%	*n*	%	*n*	%	*n*	%
Chemotherapy	3402	12.1	2116	11.1	1041	15.1	183	12.5	62	9.6
ARPI	11319	40.2	7682	40.2	2851	41.3	512	34.9	274	42.6
NSAA	12990	46.2	9055	47.3	2886	41.8	752	51.2	297	46.2
Other	433	1.5	280	1.5	122	1.8	21	1.4	10	1.6

*ARPI* Androgen Receptor Pathway Inhibitors, *NSAA* Non-Steroidal Anti-Androgens

### Sensitivity analysis adjusting for disease severity

A sensitivity analysis was conducted on a subset of 14,538 patients (10%) with complete cancer registry data to explore the relationship between race and subsequent treatment while accounting for disease severity (T stage, M stage, and Grade Group). In this cohort, the 5-year KM estimate was 88%, 84%, and 82% for NHB, Hispanics, and NHW patients, respectively (Supplemental Fig. 1). Consistent with the primary analysis, NHB patients were significantly less likely to have subsequent treatment compared to NHW patients in both univariable (HR = 0.71, 95% CI = 0.65–0.77) and multivariable (HR = 0.72, 95% CI = 0.66–0.79) analysis after adjusting for stage and grade. The previously observed lower likelihood of treatment escalation for Hispanic patients remained with nearly identical HRs, but given the smaller sample size, was no longer statistically significant in univariable (HR = 0.91, 95% CI = 0.78–1.06) or multivariable (HR = 0.95, 95% CI = 0.81–1.11) analysis **(**Supplemental Table 3**)**.

To assess whether the relationship between race and subsequent treatment varied by stage, we also stratified the multivariable analysis by M stage (M0/M1) and tested the interaction between race and M stage while adjusting for grade and other clinical variables. As evident in Supplemental Table 3, this interaction was not significant (*p* = 0.913), suggesting that the lower risk of subsequent treatment for Non-Hispanic Blacks is true regardless of metastatic or non-metastatic disease status.

## Discussion

Many patients with advanced and metastatic PC will progress to subsequent treatment after ADT initiation [10]. Whether race and ethnicity are associated with the time to subsequent treatment after 1 L ADT was previously unknown. To address this, we performed a population-level analysis using retrospective data from the nationwide VAHCS to compare racial differences (NHBs, NHWs, Hispanics, and Others) in time to subsequent treatment after 1 L ADT. Using a cohort of over 140,000 patients, we found all races were less likely to receive subsequent treatment relative to NHWs, especially among younger patients, with NHBs having the lowest subsequent treatment rates. This difference between NHBs and NHWs remained significant across all sensitivity and exploratory analyses, including those accounting for competing risks and in the subset of patients with stage and grade data available. Whether this represents better cancer control or undertreatment of patients who do progress remains to be determined.

The baseline characteristics of our population revealed notable differences across racial and ethnic groups, which may contribute to variations in treatment patterns and outcomes. For example, NHB patients were more likely to receive radiation therapy prior to ADT initiation compared to NHWs (10% vs. 7%, respectively). NHB patients were also younger at ADT initiation (median 68.1 years vs. 73.0 years for NHWs) and had higher PSA levels (median 10.4 ng/mL vs. 7.9 ng/mL), which may suggest differences in disease presentation or tumor biology. Additionally, NHB patients had the highest proportion of ≥3 comorbidities (46%), which could further influence treatment strategies and outcomes. Hispanic patients, on the other hand, had the highest proportion of individuals with a BMI of 25–29 (42%). These differences may reflect variations in lifestyles, healthcare access, or race-specific characteristics. Importantly, all of these variables were adjusted for in our multivariable analyses and thus our findings are independent of these differences. Nonetheless, understanding how these baseline factors influence treatment decisions and outcomes is critical to addressing disparities and improving care for all racial and ethnic groups.

In patients with metastatic PC and even in certain circumstances for advanced PC (localized very high risk with radiation; biochemical recurrence with short PSA doubling time), guidelines and clinical trial data universally support intensified ADT as this improves long-term outcomes. However, despite these guidelines, a subset of patients will receive ADT alone [11]. Moreover, historically (i.e. the period covered during this study), intensified ADT was not routinely recommended as the seminal studies had not yet been conducted showing the benefits of intensified ADT. Typically, PC progresses to CRPC within an average of 2–3 years, necessitating subsequent treatment to effectively manage the disease [7]. As such, subsequent treatment can be viewed as a sign that the initial treatment is no longer effective. Subsequent treatment options include but are not limited to chemotherapy, ARPIs, or NSAAs, all with the goal of slowing cancer progression, extending survival, and improving quality of life [12]. In real-world studies, where capturing disease progression is challenging, time to next treatment is often used as an intermediate endpoint [13, 14]. However, delayed subsequent treatment can reflect either better tumor control (i.e., no need for subsequent treatment) or undertreatment of patients who do progress. In large population-based claims studies, distinguishing between these possibilities is difficult. Nonetheless, subsequent treatment remains an important clinical endpoint as it signals a step-up in care with potential associated side effects. To our knowledge, no studies have specifically examined race as a prognostic factor for subsequent treatment in PC. However, this has been investigated in other malignancies. For example, Whitaker et al. found that Black patients with metastatic breast cancer were less likely to receive second-line treatment, leading to differences in overall survival. This underscores the importance of further investigating the impact of race on treatment patterns across various diseases [15].

The effectiveness of ADT in managing PC may vary across races. Vidal et al. examined the relationship between race and metastases development in men receiving ADT after non-metastatic biochemical recurrence following radical prostatectomy [10]. Outcomes were comparable between White and Black individuals, suggesting race didn’t significantly influence the risk of metastases in this population. However, the study included a modest sample size, thus findings should be interpreted with caution. Similarly, a recent systematic review of men with metastatic Castration-Sensitive Prostate Cancer (mCSPC) treated with ADT alone (with or without NSAA), found similar survival outcomes between Whites and Blacks [16]. Nonetheless, some studies found poorer survival in mCSPC for Black men vs. White [17, 18]. Notably, among the patient population newly starting ADT, no studies that we are aware of, show *better* outcomes among Black men. As such, it is intriguing that while we found that rates of subsequent treatment were lower for NHB men, especially among younger patients, the exact causes of this are unknown. Possibly, this may reflect undertreatment of Black men when they progress, as prior studies in both Medicare and the VA populations have demonstrated [11, 19, 20]. Alternatively, our results may reflect better tumor control in Black men relative to White men. While intriguing, there are no current data suggesting improved outcomes among Black men with PC treated with ADT alone vs. White men. However, our large sample size, allowed us to detect modest differences in subsequent treatment. Thus, prior studies assessing outcomes by race may have been underpowered to detect improved tumor control among Black men. Therefore, we cannot conclude with certainty whether our results reflect undertreatment and/or improved outcomes among Black men and this requires further study using other surrogate endpoints for tumor control.

Subsequent treatment rates were also lower among Hispanics and individuals from other racial or ethnic backgrounds compared to NHWs, especially among younger patients. We are not aware of data specifically examining ADT and tumor control across Hispanic and Other races. However, the same recent systematic review of men with metastatic PC found no significant difference in overall survival between Hispanics and NHW men with PC [16]. Similar to NHB men outlined above, the absence of data suggesting differences in tumor control with ADT suggests that the lower rates of subsequent treatment may reflect undertreatment of recurrent disease. Notably, prior studies, due to limited sample sizes, may not have been powered to detect the modest associations seen in our study. Intriguingly, literature suggests Black men have better outcomes with other systemic treatments for PC such as chemotherapy or novel hormonal therapies [21–23]. As such, future studies are needed to assess ADT efficacy across races.

One of the notable strengths of this study is its substantial sample size of all races, which enhances the statistical power and robustness of the findings. The inclusion of a large number of NHB individuals, often underrepresented in PC studies, improves the generalizability of the results. Moreover, a notable strength of our study is the VA’s equal access setting and low-cost medications reduce socioeconomic barriers to care.

Despite these strengths, the study has limitations. First, we were unable to isolate the precise reasons for subsequent treatments. Second, we did not capture any other measures of oncological control (PSA response, time to metastasis) which could have provided evidence to support or contradict the hypothesis that the lower rates of subsequent treatments may be linked to better cancer control. Patients were included from 2001, before current therapies (e.g., abiraterone, enzalutamide) were available, which likely explains the high rate of NSAA use as the next treatment. Finally, we lacked information on PC prognostic factors (stage, grade, disease status) to include in our multivariable models.

## Conclusions

This is the first population-level study assessing racial differences in time to subsequent treatment of men receiving 1 L ADT. We found that all races had a significantly lower rate of subsequent treatment relative to NHWs, especially among younger patients.

Reasons for this variation in practice are unknown, highlighting the need for additional research on how patients should receive timely and appropriate care throughout their PC treatment

## Supplementary information

Supplemental legends

Supplemental figures and tables

## Data Availability

The data that support the findings of this study are available from the authors upon reasonable request, within guidelines of VA rules and data sharing policies.
