# Supplementary material for: Assessing racial differences in time to subsequent treatment following androgen deprivation therapy among Veterans with prostate cancer
Source: Prostate Cancer Prostatic Dis. 2025 Jul 4;29(1):103–10. doi: 10.1038/s41391-025-00995-4 (PMC12909120; doi:10.1038/s41391-025-00995-4)
Supplement: Supplementary file 1 — Supplemental legends [file 41391_2025_995_MOESM1_ESM.docx]

**Supplemental Table 1:** Hazard ratios for the association between race and time to treatment escalation stratified by age at ADT initiation (N=141,495).

**Supplemental Table 2:** Multivariable hazard ratios for the association between race and time to subsequent treatment with year of ADT modeled with splines rather than as linear (N=141,495).

**Supplemental Table 3:** Hazard ratios for the association between race and time to treatment escalation stratified by M stage in a subset of patients with T stage, M stage, and grade available (N=14,538).

**Supplemental Figure 1.** Kaplan-Meier curve for time to subsequent treatment stratified by race among a subset of patients with stage, grade, and metastasis status available**.**
