# Supplementary material for: Assessing racial differences in time to subsequent treatment following androgen deprivation therapy among Veterans with prostate cancer
Source: Prostate Cancer Prostatic Dis. 2025 Jul 4;29(1):103–10. doi: 10.1038/s41391-025-00995-4 (PMC12909120; doi:10.1038/s41391-025-00995-4)

| **Supplemental Table 1: Hazard ratios for the association between race and time to treatment escalation stratified by age at ADT initiation (N=141,495).**  The p-value for the interaction between race and age in the multivariable model was 0.0005. | | | | | | | |
| --- | --- | --- | --- | --- | --- | --- | --- |
|  |  | **Univariable** | | | **Multivariable*** | | |
| **Group** | **Variable** | **HR** | **95% CI** | **p-value** | **HR** | **95% CI** | **p-value** |
| Age  <60 years | Race/Ethnicity |  |  |  |  |  |  |
|  | Non-Hispanic White |  | Ref. |  |  | Ref. |  |
|  | Non-Hispanic Black | 0.84 | (0.78, 0.90) | <0.001 | 0.75 | (0.69, 0.81) | <0.001 |
|  | Hispanic | 0.90 | (0.75, 1.09) | 0.279 | 0.86 | (0.71, 1.04) | 0.110 |
|  | Other | 0.83 | (0.65, 1.06) | 0.143 | 0.77 | (0.60, 0.98) | 0.031 |
| Age  60-69 years | Race/Ethnicity |  |  |  |  |  |  |
|  | Non-Hispanic White |  | Ref. |  |  | Ref. |  |
|  | Non-Hispanic Black | 0.89 | (0.85, 0.93) | <0.001 | 0.79 | (0.75, 0.82) | <0.001 |
|  | Hispanic | 1.00 | (0.91, 1.10) | 0.986 | 0.99 | (0.90, 1.09) | 0.891 |
|  | Other | 0.97 | (0.85, 1.11) | 0.646 | 0.92 | (0.80, 1.05) | 0.220 |
| Age  70-79 years | Race/Ethnicity |  |  |  |  |  |  |
|  | Non-Hispanic White |  | Ref. |  |  | Ref. |  |
|  | Non-Hispanic Black | 0.91 | (0.87, 0.96) | <0.001 | 0.83 | (0.79, 0.87) | <0.001 |
|  | Hispanic | 0.85 | (0.78, 0.93) | <0.001 | 0.90 | (0.82, 0.98) | 0.013 |
|  | Other | 0.94 | (0.82, 1.07) | 0.348 | 0.90 | (0.79, 1.03) | 0.127 |
| Age ≥80 years | Race/Ethnicity |  |  |  |  |  |  |
|  | Non-Hispanic White |  | Ref. |  |  | Ref. |  |
|  | Non-Hispanic Black | 1.07 | (0.99, 1.14) | 0.055 | 0.99 | (0.93, 1.06) | 0.838 |
|  | Hispanic | 0.90 | (0.81, 1.01) | 0.062 | 0.91 | (0.81, 1.01) | 0.080 |
|  | Other | 0.98 | (0.83, 1.17) | 0.855 | 0.95 | (0.80, 1.12) | 0.519 |
| *Multivariable model adjusted for age, year of ADT start, months from PC diagnosis to ADT start, CCI, PSA, testosterone level, BMI, and prior radiation treatment. Abbreviations: ADT= Androgen Deprivation Therapy; PSA= Prostate Specific Antigen; CCI= Charlson Comorbidity Index; BMI= Body Mass Index; PC= Prostate Cancer. | | | | | | | |

| **Supplemental Table 2: Multivariable hazard ratios for the association between race and time to subsequent treatment with year of ADT modeled with splines rather than as linear (N=141,495).** | | | |
| --- | --- | --- | --- |
| **Variable** | **HR** | **95% CI** | **p-value** |
| Race/Ethnicity |  |  |  |
| Non-Hispanic White |  | Ref. |  |
| Non-Hispanic Black | 0.83 | (0.80, 0.85) | <0.001 |
| Hispanic | 0.93 | (0.88, 0.98) | 0.006 |
| Other | 0.91 | (0.84, 0.98) | 0.016 |
| *Multivariable model adjusted for age, year of ADT start (using splines with 4 knots placed at the quartiles), months from PC diagnosis to ADT start, CCI, PSA, testosterone level, BMI, and prior radiation treatment. Abbreviations: ADT= Androgen Deprivation Therapy; PSA= Prostate Specific Antigen; CCI= Charlson Comorbidity Index; BMI= Body Mass Index; PC= Prostate Cancer. | | | |

| **Supplemental Table 3: Hazard ratios for the association between race and time to treatment escalation stratified by M stage in a subset of patients with T stage, M stage, and grade available (N=14,538).**  The p-value for the interaction between race and M stage in the multivariable model was 0.913. | | | | | | | |
| --- | --- | --- | --- | --- | --- | --- | --- |
|  |  | **Univariable** | | | **Multivariable*** | | |
| **Group** | **Variable** | **HR** | **95% CI** | **p-value** | **HR** | **95% CI** | **p-value** |
| All patients | Race/Ethnicity |  |  |  |  |  |  |
|  | Non-Hispanic White |  | Ref. |  |  | Ref. |  |
|  | Non-Hispanic Black | 0.71 | (0.65, 0.77) | <0.001 | 0.72 | (0.66, 0.79) | <0.001 |
|  | Hispanic | 0.91 | (0.78, 1.06) | 0.244 | 0.95 | (0.81, 1.11) | 0.485 |
| M0  No Metastasis | Race/Ethnicity |  |  |  |  |  |  |
|  | Non-Hispanic White |  | Ref. |  |  | Ref. |  |
|  | Non-Hispanic Black | 0.73 | (0.66, 0.80) | <0.001 | 0.73 | (0.66, 0.80) | <0.001 |
|  | Hispanic | 0.89 | (0.75, 1.06) | 0.178 | 0.95 | (0.80, 1.14) | 0.593 |
| M1  Metastasis | Race/Ethnicity |  |  |  |  |  |  |
|  | Non-Hispanic White |  | Ref. |  |  | Ref. |  |
|  | Non-Hispanic Black | 0.76 | (0.62, 0.93) | 0.008 | 0.73 | (0.59, 0.91) | 0.005 |
|  | Hispanic | 0.77 | (0.56, 1.06) | 0.113 | 1.04 | (0.75, 1.46) | 0.809 |
| *Multivariable model adjusted for age, year of ADT start, months from PC diagnosis to ADT start, CCI, PSA, testosterone level, BMI, prior radiation treatment, metastasis, T stage, and Grade Group. Abbreviations: ADT= Androgen Deprivation Therapy; PSA= Prostate Specific Antigen; CCI= Charlson Comorbidity Index; BMI= Body Mass Index; PC= Prostate Cancer. | | | | | | | |

**Supplemental Figure 1.** **Kaplan-Meier curve for time to subsequent treatment stratified by race among a subset of patients with stage, grade, and metastasis status available**


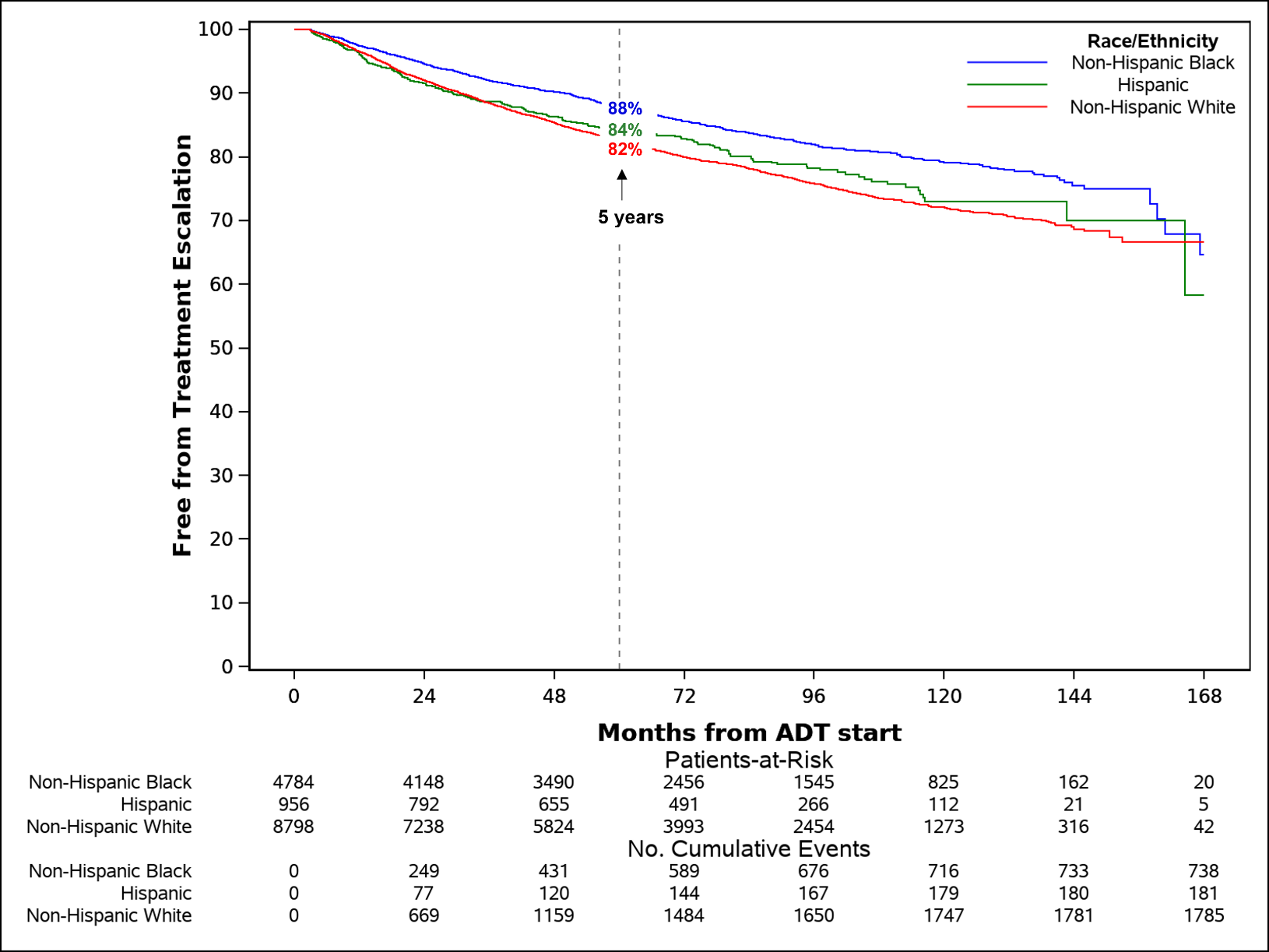

Supplement: Supplementary file 2 — Supplemental figures and tables [file 41391_2025_995_MOESM2_ESM.docx]
